# Gold Nanocage-Based Electrochemical Sensing Platform for Sensitive Detection of Luteolin

**DOI:** 10.3390/s18072309

**Published:** 2018-07-17

**Authors:** Xiaobao Li, Ruyi Zou, Yanyan Niu, Wei Sun, Taiming Shao, Xiaoqin Chen

**Affiliations:** 1 Key Laboratory of Tropical Medicinal Plant Chemistry of Ministry of Education, College of Chemistry and Chemical Engineering, Hainan Normal University, Haikou 571158, China; lixiaobao0797@163.com (X.L.); zoury@sru.jx.cn (R.Z.); niuyanyan1986@126.com (Y.N.); cxq2019@126.com (X.C.); 2 Key Laboratory of Sensor Analysis of Tumor Marker of Ministry of Education, College of Chemistry and Molecular Engineering, Qingdao University of Science and Technology, Qingdao 266042, China; 3 Key Laboratory of Medicinal and Edible Plants Resources of Hainan Province, School of Chemical and Material Engineering, Hainan Institute of Science and Technology, Haikou 571126, China; shaotaiming@sina.com

**Keywords:** luteolin, gold nanocages, carbon ionic liquid electrode, electrochemistry

## Abstract

A simple and sensitive electrochemical sensor was developed for the detection of tracelevels of luteolin. The sensoris based on a novel type of chemically modified electrode: gold nanocage (AuNCs)-modified carbon ionic liquid electrode (CILE). To construct this electrochemical sensing platform for luteolin, CILE is initially prepared by using 1-hexylpyridinium hexafluorophosphate as the binder and then AuNCs are coated on the surface of CILE to fabricate AuNCs-modified CILE (AuNCs/CILE). Electrochemical studies have shown that AuNCs/CILE can exhibit enhanced electrocatalytic activity toward the redox reaction of luteolin, therefore, the redox peak current of luteolin can be greatly improved, resulting in the high sensitivity of the developed sensor. Under the optimal conditions, the oxidation peak currents of the sensor increase linearly with an increase in the luteolin concentration in a range from 1 to 1000 nM with a detection limit of 0.4 nM, which is lower than those of most reported electrochemical luteolin sensors. Moreover, the reproducibility, precision, selectivity, and stability of this sensor are excellent. Finally, the sensing system was applied to the analysis of luteolin-spiked drug samples and the recovery in all cases was 95.0–96.7%, indicating the potential application of this simple, facile, and sensitive sensing system in pharmaceutical analysis.

## 1. Introduction

Luteolin (3′,4′,5,7-tetrahydroxyflavone) is an important flavonoid that is naturally present in a variety of plants, e.g., peppermint, green pepper, thyme, and parsley [1,2,3]. In clinical applications, luteolin can be used as a drug to treat many diseases (e.g., respiratory disease, cardiovascular disease, and hyperlipidemia). This is because it has unique biological functions and pharmacological activities, e.g., anti-inflammatory, anti-allergic, anti-ulcer, and anti-oxidation effects, anti-cancer and anti-viral activities, cardiovascular protection, and cataract prevention [4,5,6,7,8]. Moreover, recent studies have demonstrated that luteolin can enter the cell nucleus and suppress oxidative damage of deoxyribonucleic acid [9]. Consequently, the detection and monitoring of luteolin in pharmaceuticals and biological fluids is of great importance for drug quality control, clinical medicine research, and biochemical study.

Up to now, many analytical methods have been developed for the detection of luteolin. These have been basedon various techniques, including high-performance liquid chromatography, gas chromatography, mass spectrometry, capillary electrophoresis, and spectrophotometry [10,11,12,13,14,15]. Although these methods show high sensitivity and selectivity for detecting luteolin, most of them require expensive and sophisticated instruments, complicated operations, and professional operators, which limit their practical applications. In contrast, electrochemical methods are considered as simple, cost-effective, and sensitive techniques for luteolin detection because they can be easily carried out with inexpensive and simple instruments [4,5,16,17,18,19,20,21]. Liu et al. investigated the electrochemical behavior of luteolin at a glassy carbon electrode (GCE), based on which they developed an electrochemical sensor for the detection of luteolin with a detection limit of 5.0 nM [4]. To improve the sensitivity of electrochemical detection of luteolin, recent research has focused on the design of innovative, chemically modified electrodes based on various nanomaterials, such as multi-walled carbon nanotubes, macroporous carbon nanomaterials, graphene-hydroxyapatite nanocomposites, and In_2_O_3_ nanoparticles [5,16,17,18,19,20]. These nanomaterials exhibit excellent electrocatalytic activity toward the redox reaction of luteolin and lead to the amplification of the electrochemical signal. For example, Guo’s group reported a sensitivity-enhanced electrochemical sensor for determining luteolin using macroporous carbon nanomaterial modified GCE with a detection limit of 1.3 nM [17]. Along with the development of nanoscience, it is still necessary to exploit new nanomaterials with high electrocatalytic activity for further improving the sensitivity of electrochemical luteolin sensors.

Gold nanocages (AuNCs), representing an emerging class of nanosized gold material, have attracted much research interests since being invented by Xia’s group in 2002 [22,23,24,25]. Due to the unique features of noble metal composition with hollow, porous, and thin-walled structure, AuNCs possess several distinctive properties over commonly used gold nanoparticles, including good chemical/thermal stability, high catalytic activity, strong localized surface plasmon resonance, and excellent controlled release properties [22,23,24,25]. Such outstanding properties make them very attractive for many applications including catalysis, diagnostics, therapy, and spectral signal enhancement, which enable the constructed devices/methods with unparalleled performance [22,23,24,25]. For example, by using the reduction of *p*-nitrophenol by NaBH_4_ as a model reaction, Xia et al. demonstrated that AuNCs are catalytically more active than solid gold nanoparticles [26]. The advantages of AuNCs inspired us to investigate whether it is possible to utilize them as the electrocatalyst to design a new chemically-modified electrode for luteolin sensing. To the best of our knowledge, there has been no report about the employment of AuNCs for the development of electrochemical luteolin sensors.

Herein, we design a new sensitive electrochemical sensor for the determination of luteolin using AuNCs-modified carbon ionic liquid electrode (CILE) as a sensing platform (AuNCs/CILE). CILE is prepared by using 1-hexylpyridiniumhexafluorophosphate (HPPF_6_) as the binder, which has been widely reported as the substrate electrode for electroanalysis [27,28]. CILE has been proven to have advantages such as high conductivity, anti-fouling ability, and good stability, which are due to the use of high conductive ionic liquid (IL) as the binder and the modifier. The assay is performed by utilizing AuNCs/CILE as the working electrode via a voltammetric method. The electrochemical signal of luteolin can be recorded because luteolin is an electroactive compound [4,5]. By monitoring the change in the electrochemical signal, we could quantitatively determine the concentration of target luteolin in samples. Notably, the employment of AuNCs as the electrocatalyst in this sensing system endows AuNCs/CILE with excellent electrocatalytic activity toward the redox reaction of luteolin with high sensitivity for luteolin detection. The study aims to emphasize that AuNCs with superior electrocatalytic activity can be utilized as an alternative to previously used nanocatalysts in electrochemical sensing of luteolin.

## 2. Experimental

### 2.1. Reagents

Luteolin (≥99%), baicalein (≥99%), and quercetin (≥99%) were purchased from Xi’an Yuquan Biotechnology Co. Ltd. (Xi′an, China). Graphite powder (average particle size 30 μm, >99%) was obtained from Shanghai Huayi Group Huayuan Chem. Industry Co. Ltd. (Shanghai, China). Paraffin liquid was obtained from Tianjin Damao Chemical Reagent Factory (Shanghai, China). HPPF_6_ (≥99%) was obtained from Lanzhou Yulu Fine Chemical Co. Ltd. (Lanzhou, China). AuNCs (50 µg/mL) were purchased from Nanjing XFNANO Materials Tech. Co. Ltd. (Nanjing, China). All other reagents and chemicals were of analytical grade at least. All aqueous solutions were prepared using deionized (DI) water (18.2 MΩ·cm) obtained from a Milli-Q water purification system (Millipore, Burlington, MA, USA). Phosphate buffered saline (PBS) was used as electrolyte and different pH values were prepared by mixing stock solutions of 0.1 M NaH_2_PO_4_ and 0.1 M Na_2_HPO_4_, and then the required pH values were adjusted by using 0.1 M H_3_PO_4_and 0.1 M NaOH.

### 2.2. Apparatus

All electrochemical measurements including cyclic voltammetry (CV) and differential pulse voltammetry (DPV) were performed on a CHI 1220B electrochemical workstation (Shanghai CH Instrument, Shanghai, China). The electrochemical impedance spectroscopy (EIS) was performed on a CHI 750B electrochemical workstation (Shanghai CH Instrument, Shanghai, China). A conventional three-electrode system was used with AuNCs/CILE as the working electrode, a platinum wire electrode as the auxiliary electrode, and a saturated calomel electrode (SCE) as the reference electrode. Scanning electron microscopy (SEM) was conducted with a JSM-7100F scanning electron microscope (JEOL, Tokyo, Japan) and transmission electron microscopy (TEM) on a JEM-2010F transmission electron microscope (JEOL, Tokyo, Japan).

### 2.3. Fabrication of AuNC/CILE

In a standard fabrication procedure, CILE was initially prepared as follows [27,28]. First, 4.8 g of graphite powder, 2.4 g of HPPF_6_, and 1500 µL of liquid paraffin were mixed and heated at 80 °C for 1 h to form a homogeneous carbon paste. A portion of the formed carbon paste was then packed into one end of a glass tube that had a diameter of 4 mm, and a copper wire was inserted through the other end of the tube to establish an electrical contact. Before use, the electrode surface was polished on a weighing paper to obtain a mirror-like surface. Then, 8 µL of 50 µg/mL AuNCs water solution was dropped on the surface of CILE. After being dried at room temperature, the fabricated AuNCs/CILE was stored in a refrigerator at 4 °C for future use.

## 3. Results and Discussion

### 3.1. Material Characterization

**Figure 1:**
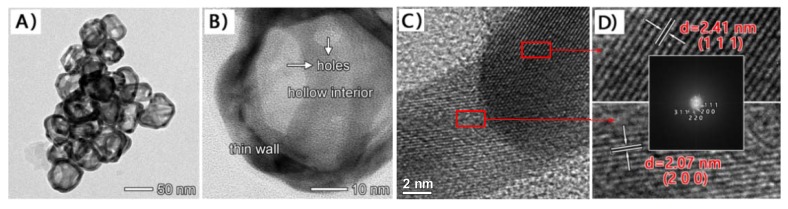
(**A**) Regular TEM image; (**B**,**C**) HRTEM image of an individual AuNC; (**D**) HRTEM under amplification (insert is fast-Fourier transform electron diffraction pattern).

Figure 1A shows a typical TEM image of the AuNCs, which displayed well-defined hollow shapes with thin walls and holes on the surfaces. The average external diameter and wall thickness of the hollow particles were measured to be 54.3 nm and 5.4 nm, respectively, with narrow size distributions. The high-resolution TEM (HRTEM) image of an individual particle (Figure 1B) further clearly shows the features of the hollow interiors, thin walls, and porous surfaces for AuNCs. These TEM results demonstrate that the AuNCs used were of high quality, thereby forming the basis for the construction of the subsequent electrochemical sensor. According to the fast-Fourier transform electron diffraction pattern and XRD standard card {PDF#04–0784}, we revealed that AuNCs belonged to {111}, {200}, {220}, and {311} crystal forms based on its lattice fringe separations, and the nanomaterial mainly presented {111} and {200} crystal faces whose lattice fringe separations were calculated as 2.41 nm and 2.07 nm, respectively.

**Figure 2:**
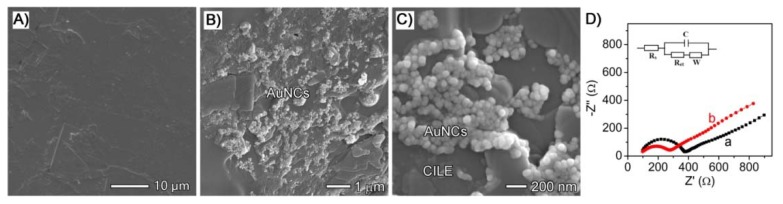
SEM images of a carbon ionic liquid electrode (CILE) (**A**) and AuNCs/CILE with different magnification (**B**,**C**); (**D**) Nyquist plots of CILE (a) and AuNCs/CILE (b) in a 10.0 mmol/L [Fe(CN)_6_]^3−/4−^ solution containing 0.1 mol/L KCl with the frequency of 10^5^~0.1 Hz (inset is the Randles circuit model in the cell).

Figure 2 shows the SEM images of different interfaces of working electrodes. As shown in Figure 2A, CILE appeared flatly and the void spaces of graphite powders were connected by ionic liquid (IL) with high viscosity to verify the successful preparation of CILE. The surface of the as-fabricated AuNCs/CILE was characterized using SEM. Figure 2B,C show representative low- and high-magnification SEM images of AuNCs/CILE surface prepared from a standard fabrication. It can be seen that the surface of CILE displayed uniform morphology without separated carbon layers, which was similar to previous reports [27,28], and the AuNCs were well dispersed on the surface of the CILE. These results indicate the successful preparation of AuNCs/CILE based on the standard fabrication process.

Further, EIS was used to investigate the impedance behaviors of AuNCs/CILE. Figure 2D shows the Nyquist plots of CILE and AuNCs/CILE in a 10.0 mmol/L [Fe(CN)_6_]^3−/4−^ solution containing 0.1 mol/L KCl with the frequency of 10^5^~0.1 Hz. The Randles circuit was used to fit the impedance data obtained in the experiment with the scheme added as the insert of Figure 2D, where *Rs* is solution resistance, *C* is interface capacitance, *Ret* is electron transfer resistance, and *W* is dispersion resistance. The resistance of AuNCs/CILE was much lower than that of CILE, indicating that the presence of AuNCs on the surface of CILE could enhance the interfacial conductivity of the electrode and accelerate the electron transfer, which could be attributed to high conductivity with the hollow and porous nanostructure of AuNCs. Therefore, AuNCs could greatly improve the electrochemical performance of the modified electrode. 

### 3.2. Direct Electrochemical Behavior of Luteolinon the Modified Electrode

**Figure 3:**
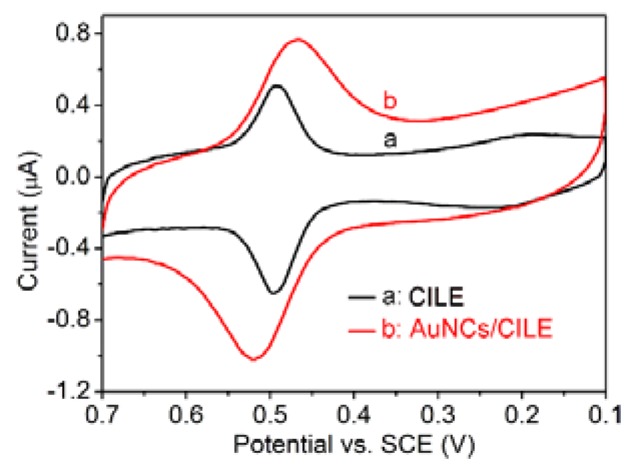
Cyclic voltammograms of 1.0 µM luteolin on CILE (a) and AuNCs/CILE (b) in 0.1 M PBS (pH = 3) at a scan rate of 100 mV/s.

To investigate the electrochemical behavior of luteolin on AuNCs/CILE, we carried out CV to record the cyclic voltammogram of 1.0 µM luteolin on AuNCs/CILE. As shown in Figure 3, a pair of well-defined redox peaks could be obtained on AuNCs/CILE (curve b) for the luteolin solution. The cathodic and anodic peak currents were measured to be I_pc_ = 0.4688 μA and I_pa_ = 0.7076 μA, respectively. The results indicate that the redox reaction of luteolin had taken place on AuNCs/CILE, which was in line with the electrochemical reaction mechanism of luteolin on electrodes [4,5]. For comparison, we also recorded the cyclic voltammogram of 1.0 µM luteolin on CILE under the same conditions, and the corresponding anodic and cathodic peak currents were I_pc_ = 0.3054 μA and I_pa_ = 0.5145 μA, respectively (curve a in Figure 3). The redox peak currents of luteolin on AuNCs/CILE were about 1.5-fold higher than those on CILE, indicating the superiority of AuNCs/CILE. The reason could be that AuNCs have good conductivity and a hollow and porous nanostructure, which could increase the active surface area of the electrode and improve the electron transfer kinetics. By using the oxidation peak currents as the detection signal, an electrochemical sensing platform for the determination of luteolin could be provided. However, the peak-to-peak separation (ΔE_p_) of luteolin on CILE was 3.0 mV, which was smaller than that of AuNCs/CILE (55.0 mV). The results may be due to the porous AuNCs with a large surface area, which exhibited certain adsorption activity and decreased the diffusional rate of luteolin from solution to electrode.

### 3.3. Electrochemical Investigations

**Figure 4:**
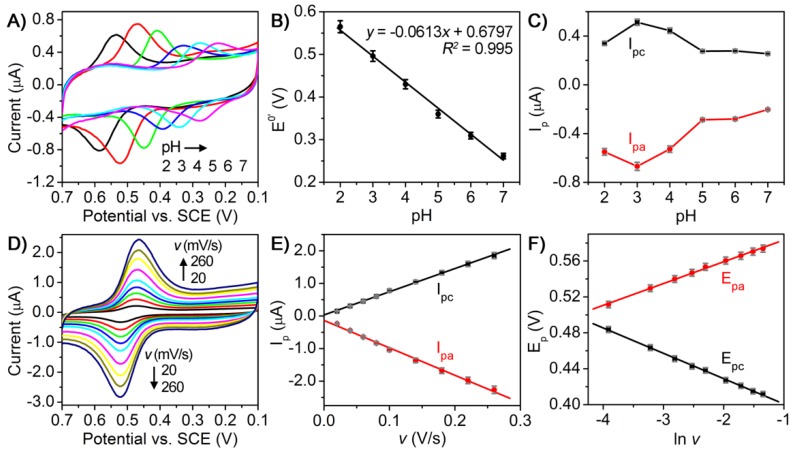
(**A**) Cyclic voltammograms of 1.0 µM luteolin on AuNCs/CILE in PBS with different pH values (2, 3, 4, 5, 6, 7) at a scan rate of 100 mV/s; (**B**) Relationship between E^0^′ and pH; (**C**) Relationship between I_p_ and pH; (**D**) Cyclic voltammograms of 1.0 µM luteolin on AuNCs/CILE in 0.1 M PBS (pH = 3) at different scan rates (*v*; 20, 40, 60, 80, 100, 140, 180, 220, 260 mV/s); (**E**) Linear relationship between I_p_ and *v*; (**F**) Linear relationship between E_p_ and ln*v*.

To gain more insight into the electrochemical behavior of luteolin on AuNCs/CILE, we also conducted a set of experiments to study the influence of buffer pH and scan rate on cyclic voltammogram of luteolin on the electrode. Figure 4A shows the cyclic voltammetric responses of 1.0 µM luteolin on AuNCs/CILE in the pH range of 2.0~7.0. By plotting the formal potentials (E^0^′) and the redox peak currents (I_p_) against buffer pH values, the relationship between E^0^′ and pH (Figure 4B) and I_p_ and pH (Figure 4C) could be obtained, respectively. It can be found that the redox peak potential shifted negatively as the buffer pH increased from 2.0 to 7.0, suggesting the participation of protons in the electrode reaction process (Figure 4A,B). More importantly, a linear dependence between the E^0^′ and pH was obtained with a slope value of −61.3 mV/pH that was close to the theoretical value of −59 mV/pH, indicating that the redox reaction of luteolin on the electrode involves an equal number of electron and proton transfers (Figure 4B) [29]. In addition, the I_p_ value increased rapidly with the increase of pH from 2.0 to 3.0 and decreased gradually after pH = 3, revealing that the electrode reaction could more easily proceedin the acidic solution with more protons (Figure 4C). The maximum I_p_ value appeared at pH = 3.0 and, thus, pH 3.0 was used as the optimal pH in the following experiments.

**Figure 5:**
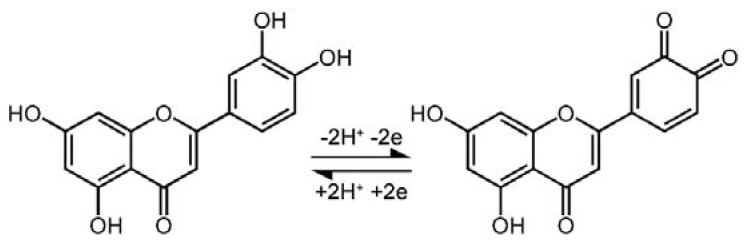
Electrochemical reaction mechanism of luteolin.

Figure 4D shows the cyclic voltammetric responses of 1.0 µM luteolin on AuNCs/CILE at various scan rates ranging from 20 to 260 mV/s. An increase in the scan rate resulted in a gradual increase in the redox peak currents, together with a positive shift of the anodic peak and a negative shift of the cathodic peak. By plotting the redox peak currents (I_p_) against the scan rates (*v*), two good linear relationships between I_p_ and *v* were observed with the regression equations of I_pa_ (µA) = −8.41*v* (V/s) − 0.136 (*R*^2^ = 0.996) and I_pc_ (µA) = 7.12*v* (V/s) + 0.0312 (*R*^2^ = 0.999), indicating an adsorption-controlled electrode reaction process (Figure 4E). Furthermore, the plots between the redox peak potentials (E) and the natural logarithm of scan rates (ln*v*) also exhibited two good linear relationships with the regression equations of E_pa_ (V) = 0.0243ln*v* + 0.608 (*R*^2^ = 0.998) and E_pc_ (V) = −0.0282ln*v* + 0.373 (*R*^2^ = 0.999). According to Laviron’ smodel, the slopes of the equations for E_pa_ − ln*v* and E_pc_ − ln*v* can be expressed as RT/(1 − α)nF and −RT/αnF, respectively. Note that R represents the gas constant, α is the electron transfer coefficient, n is the electron transfer number, and F is Faraday’s constant [30]. The values of α and n were calculated to be 0.463 and 1.97, respectively. Therefore, the number of electrons involved in the redox reaction of luteolin on AuNCs/CILE was found to be 2. All the above results demonstrate that the electrochemistry of luteolin on AuNCs/CILE was a two-proton two-electron process with the reaction mechanism shown in Figure 5.

### 3.4. Analytical Performance

**Figure 6:**
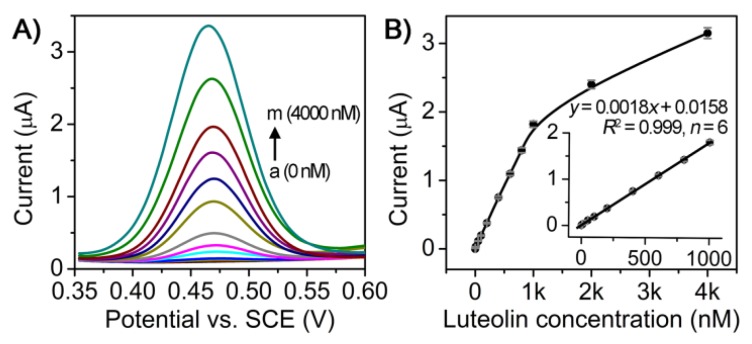
Detection of luteolin standard solution using AuNCs/CILE-based electrochemical sensor. (**A**) DPV curves of different concentrations of luteolin standards on AuNCs/CILE from a tom as 0, 0.001, 0.005, 0.01, 0.05, 0.1, 0.2, 0.4, 0.6, 0.8, 1.0, 2.0, 4.0 μmol/L; DPV parameters were as follows: pulse amplitude as 0.05 V; pulse width as 0.02 s; pulse period as 0.2 s; and quiet time as 0.5 s; (**B**) Calibration curve generated by plotting the oxidation peak current as a function of luteolin concentration. Inset showed the linear range region of the calibration curve.

On the basis of the above investigation, we evaluated the analytical performance of the AuNCs/CILE-based electrochemical sensor for detecting luteolin by using DPV. The sensitivity and the dynamic working range of this sensor were first assessed against a luteolin standard solution with various concentrations. Figure 6A shows the DPV responses of the sensor: the DPV oxidation peak currents increased with the increment of luteolin concentrations. A quality linear correlation (*R*^2^ = 0.999) between the DPV oxidation peak current and the concentration of standard luteolin solution could be obtained in the range of 1–1000 nM with a low limit of detection (LOD) of 0.4 nM estimated using 3σ (where σ is the standard deviation of a blank solution, *n* = 6, Figure 6B). As shown in Table 1, the LOD value was lower than most of the reported electrochemical methods for luteolin detection. The results indicate the high sensitivity of the developed sensor, which could be attributed to the enhanced electrocatalytic activity of AuNCs/CILE.

The reproducibility and precision of this sensor were monitored by repeatedly assaying 10, 100, and 1000 nM luteolin standard solutions as examples. Experimental results indicate that the relative standard deviations (RSDs, *n* = 6) of the intra-assay using identical batches of AuNCs/CILE were 0.7%, 1.1%, and 1.9% for 10, 100, and 1000 nM luteolin, respectively. The RSDs of the interassay with various batches were 1.2%, 1.8%, and 2.4% (*n* = 6), respectively, toward the concentrationsmentioned above. The low RSDs suggest an excellent precision and reproducibility of the developed sensor.

The selectivity of the sensor was examined by challenging the sensing system with some other possible interfering substances that may be present in pharmaceutical and biological samples, e.g., Na^+^, K^+^, Ba^2+^, Cd^2+^, Mn^2+^, Co^2+^, lysine, threonine, glycine, and alanine. Specifically, the electrochemical response of 1 µM luteolin was detected in the presence of 50 µM of interfering substance under optimal conditions. Note that 1 µM luteolin in pH 3.0 PBS was used as the control. As summarized in Table 2, the changes in the peak current for all interfering substances were less than ±5% relative to the control, clearly indicating that these interfering substances had no significant influence on the detection and revealing a good selectivity of the developed sensor for luteolin detection. The inference of some flavonoids such as baicalein and quercetin was also checked. Baicalein and quercetin (50 µM each) were mixed with 1 µM luteolin and the relative deviation was 2.97% and 4.24%, respectively, indicating that baicalein and quercetin with similar structures did not interfere with luteolin detection due to the different redox peak potential.

Moreover, the storage stability of the sensor was testedand the results show that the DPV oxidation peak of luteolin decreased by only 3.48% (compared with the original test) when the AuNCs/CILE had been stored in a refrigerator at 4 °C for 4 weeks, confirming good storage stability of the sensor. These results demonstrate that AuNCs/CILE could be successfully applied for the electrochemical sensing of luteolin with good analytical performance.

### 3.5. Monitoring of Real Samples

To evaluate the potential use of the proposed sensor for testing real samples, we applied it to determine luteolin in the Duyiwei capsule (Z109700–53), which was purchased from Gansu Duyiwei Pharmaceutical Ltd. Co. (Kangxian, Gansu, China). Initially, 10 capsules were dissolved with 10 mL ethanol under ultrasonication for 1 h followed by filtration. Then, the extract was diluted with 40 mL PBS (pH 3.0) to prepare the sample matrix. Subsequently, the standard addition method was performed with the following steps: (1) the sample matrix was spiked with luteolin standard to obtained luteolin-spiked drug samples with different concentrations; (2) the luteolin-spiked drug samples were analyzed using the standard procedure; (3) luteolin concentration in each sample was quantified on the basis of the calibration curve shown in Figure 6B, and (4) the recovery for each sample was calculated. The detection results are summarized in Table 3. The recoveries for all the samples ranged from 95.0% to 96.7% and RSDs for all the samples were below 2% (*n* = 3). These data strongly validated that the proposed sensor could be considered as an accurate and reliable method for the detection of luteolin in real samples.

## 4. Conclusions

In summary, we have demonstrated a novel and facile electrochemical method for sensitive detection of luteolin based on gold nanocage-modified carbon ionic liquid electrode (AuNCs/CILE) as the sensing platform. Compared with previously reported electrochemical methods for luteolin detection, the highlight of the present work is its high detection sensitivity, which relies on the enhanced electrocatalytic activity of AuNCs/CILE toward the redox reaction of luteolin. In addition to the high sensitivity, the electrochemical method also features high selectivity and precision, simple operation, and speed. We believe the method reported here will find widespread application in monitoring and quantifying luteolin in pharmaceutical scenarios. Future work would be focused on searching for new nanomaterials with highly efficient electrocatalytic activity in order to further improve the signal of the electrochemical responses.

## Figures and Tables

**Table 1:** Comparison of analytical performances of various electrochemical methods for luteolin.

Electrodes	Linear Range (nmol/L)	LOD (nmol/L)	Refs
GCE	10~1.0 × 10^3^	5	[4]
PDDA-RGO-modified GCE	1~1.0 × 10^4^	1	[5]
MWNTs-modified GCE	0.2~3	0.06	[16]
MPC-modified GCE	3.0 × 10^2^~3.0 × 10^4^	1.3	[17]
GNs-HA-modified GCE	20~1.0 × 10^4^	10	[18]
In_2_O_3_-nanoparticle-modified GCE	9.98~88.4	0.199	[19]
Nbim/CNT-modified GCE	5~320	0.6	[20]
heated pencil lead disk electrode	4~1.0 × 10^4^	1	[31]
AuNCs/CILE	1~1.0 × 10^3^	0.4	This work

**Table 2:** Summary of the detection of 1 µM luteolin in the presence of 50 µM interfering substances.

Interfering Substances	Concentration (µM)	Relative Deviation (%)
Na^+^	50	0.74
K^+^	50	3.32
Ba^2+^	50	−2.31
Cd^2+^	50	4.90
Mn^2+^	50	3.79
Co^2+^	50	3.55
Lysine	50	4.39
Threonine	50	2.30
Glycine	50	0.98
Alanine	50	3.18
Leucine	50	0.56
Baicalein	50	2.97%
Quercetin	50	4.24%

**Table 3:** Detection of luteolin in Duyiwei capsule using the standard addition method (n = 3).

Sample No.	Spiked (µM)	Found (µM)	RSD (%)	Recovery (%)
1	0	0.99	1.44	No application
2	0.2	1.18	1.32	95.0
3	0.4	1.37	1.97	95.0
4	0.6	1.57	1.62	96.7

## Abbreviations

GCE	glassy carbon electrode
GNs	Graphenenano sheets
PDDA-RGO	Poly (diallyldimethylammonium chloride) functionalized reduced graphene oxide
MWNTs	multi-walled carbon nanotubes
MPC	macroporous carbon
HA	hydroxyapatite
Nbim	nitro-substituted3,3′-bis (indolyl) methane
PBS	phosphate buffer solutions
CNT	carbon nanotube
HPPF_6_	1-butylpyridinium hexafluorophosphate
